# Collodion in Small Pox

**Published:** 1851-01

**Authors:** S. B. Brinberhoff

**Affiliations:** Pa.


### ARTICLE V.

*Application of Collodion in Small Pox, to prevent pitting.* By S. B. Brinberhoof, M. D., of Pa.

Dr. Flint—*Dear Sir:* Having pursued a treatment of my own, as far as I know, (yet it may have been practiced by others, and not have met my notice,) in two cases of small pox, I have thought perhaps it would not be doing wrong to state the facts to you. I regret that the notes I took of the case at the time, have by accident been misplaced, and I shall have to state the facts from memory. The first case occurred to a person who had never been vaccinated, to his knowledge. The second person had been vaccinated, but I could not discover any scar as showing it had worked well, or at all. I will not state particulars as to the cases, more than that I considered them both cases of virulent small pox.

The treatment was as follows: As soon as the eruption showed itself, I applied, with a fine varnish brush, to the face and neck, a coating of collodion, except the upper eye-lids, and a small place on the neck. The place on the neck I left to see whether there would be any scars, should the patient recover. I applied the mixture every day for four days, then every second or third day, as I thought required. One on the twenty-first, the other on the twenty-third day, was discharged as convalescent, with directions, should any scattering eruptions appear, to apply the mixture over them. There was scarcely a single scar on either patient, over the parts where the mixture was applied, but in each case, where there was no collodion, used, as the spot on the neck and other parts, was pitted as much as I ever saw in my life.

I pursued this practice from a suggestion in your lectures, while upon this disease, stating that covering the face, &c., with gold leaf, had been considered by some as a preventive against scars being left, by excluding the air from the affected part. The gold leaf is an expensive mode of treatment, while the collodion is cheap, and easy of application.

Should you think this worthy of notice in your Journal, you are welcome to do so, if not, just as well.—*Buffalo Medical Journal.*

